# MiR-9-3p regulates the biological functions and drug resistance of gemcitabine-treated breast cancer cells and affects tumor growth through targeting MTDH

**DOI:** 10.1038/s41419-021-04145-1

**Published:** 2021-09-22

**Authors:** Yike Wang, Lifeng Dong, Fang Wan, Fangfang Chen, Dianlei Liu, Deqin Chen, Jingpei Long

**Affiliations:** Department of Surgery, Women’s Hospital School of Medicine Zhejiang University, Hangzhou, Zhejiang China

**Keywords:** Cancer, Cell biology

## Abstract

This study explored the role of MTDH in regulating the sensitivity of breast cancer cell lines to gemcitabine (Gem) and the potential miRNAs targeting MTDH. The expression of MTDH in cancer tissues and cells was detected by immunohistochemical staining or qRT-PCR. The target genes for MTDH were predicted by bioinformatics and further confirmed by dual-luciferase reporter assay and qRT-PCR. Cancer cells were transfected with siMTDH, MTDH, miR-9-3p inhibitor, or mimics and treated by Gem, then CCK-8, colony formation assay, tube formation assay, flow cytometry, wound healing assay, and Transwell were performed to explore the effects of MTDH, miR-9-3p, and Gem on cancer cell growth, apoptosis, migration, and invasion. Expressions of VEGF, p53, cleaved caspase-3, MMP-2, MMP-9, E-Cadherin, N-Cadherin, and Vimentin were determined by Western blot. MTDH was high-expressed in cancer tissues and cells, and the cells with high-expressed MTDH were less sensitive to Gem, while silencing MTDH expression significantly promoted the effect of Gem on inducing apoptosis, inhibiting cell migration, invasion, and growth, and on regulating protein expressions of cancer cells. Moreover, miR-9-3p had a targeted binding relationship with MTDH, and overexpressed miR-9-3p greatly promoted the toxic effects of Gem on cancer cells and expressions of apoptosis-related proteins, whereas overexpressed MTDH partially reversed such effects of overexpressed miR-9-3p. The study proved that miR-9-3p regulates biological functions, drug resistance, and the growth of Gem-treated breast cancer cells through targeting MTDH.

## Introduction

Breast cancer has been increasing since the late 1970s, and gradually became a female malignancy with the highest incidence and the most frequent cause of cancer-related death [1]. At present, chemotherapy plays an important role in the systemic treatment of breast cancer, but chemotherapy resistance developed during treatment is still the main cause leading to failure of treatment and poor prognosis [2]. Therefore, it is necessary to discover effective biological indicators and molecular targets related to the prognosis of breast cancer patients, so as to provide patients with a more effective treatment.

MTDH is a metastatic adhesion factor/protein, alternatively known as astrocyte elevated gene-1 (AEG-1) [3]. High-expressed MTDH in many malignant tumors such as breast cancer, liver cancer, prostate cancer, and glioma has been found to be related to cancer prognosis [4–7]. MTDH is also involved in major malignant behaviors of tumor cells including in promoting tumor metastasis, invasion, angiogenesis, tumor cell proliferation, and growth [8]. MicroRNAs (miRNAs) are known as a class of evolutionarily conserved non-coding RNAs with ~19–25 nucleotides in length, and can bind to each other through complementary pairing with the 3′UTR sequence of the target mRNA to inhibit protein translation or degradation [9]. This study predicted that miR-9-3p can bind to MTDH by bioinformatics analysis.

Previous studies confirmed that aberrant expression of miR-9-3p is closely related to tumorigenesis, but the exact biological effects of miR-9-3p vary in different tumors [10]. Zhang et al. showed that miR-9-3p functions as an oncogene in papillary thyroid carcinoma [11]; Meng et al. found that overexpression of miR-9-3p inhibits the invasion of gastric cancer cells, and their further transcriptome analysis showed that miR-9-3p can serve as a novel tumor suppressor gene in gastric cancer [12]; miR-9-3p is abnormally expressed in breast cancer tissues [13]. However, whether miR-9-3p regulates the occurrence and development of breast cancer has not been investigated. Currently, the present study was the first to report that miR-9-3p could bind to MTDH to regulate the pathogenesis of breast cancer, and that there is a shared mechanism through which miR-9-3p and MTDH jointly regulate chemoresistance of breast cancer patients. In this study, we further explored the regulatory mechanism of miR-9-3p and MTDH in promoting the toxicity of gemcitabine (Gem) on cancer cells by performing in vitro cell experiment and in vivo mouse experiment. And the study results found that miR-9-3p can inhibit the biological function of breast cancer cells regulated by the MTDH gene and affect the growth and drug resistance of breast cancer cells.

## Methods

### Bioinformatics analysis

TCGA website was used to analyze the relationship expression of MTDH in tissues of cancer patients. Kaplan–Meier survival analysis was applied to analyze the survival prognosis of the cancer patients. The MTDH copy number of cancer tissue samples was analyzed using the GISTIC 2.0 software on the GenePattern public server platform (http://genepattern.broadinstitute.org/). Targetscan 7.2 (http://www.targetscan.org/vert_72/) and miRDB (http://mirdb.org/) were used to predict the target miRNA of MTDH.

### Clinical specimens

The breast cancer tissue and adjacent tissue samples were obtained from 85 cases of breast cancer patients who attended Women’s Hospital School of Medicine Zhejiang University for treatment between May 2017 and January 2019. The tissues were stored in liquid nitrogen at −80 °C. Our study was approved by the Ethics Committee of the Women’s Hospital School of Medicine Zhejiang University, and all the patients agreed that their tissues would be used for experimental purposes and signed a written informed consent form.

### Treated cell

Breast cancer cell lines (T47D, BT474, MCF7, HCC1806, MDA-MB-231, and BT-549 cell lines) and HEK293T cells were purchased from American Type Culture Collection (Manassas, USA), and all were cultured in Dulbecco’s modified eagle medium containing 10% fetal bovine serum (FBS, Gibco, USA) at 37 °C in a 5% CO_2_ atmosphere. The cells were treated by different concentrations (0.25, 0.5, 1, 5, and 10 nM) of Gem (CAS: 95058-81-4, molecular formula: C9H11F2N3O4, purity: 98%) at 37 °C in a 5% CO_2_ atmosphere. As MCF7 was the least sensitive to Gem, while HCC1806 was the most sensitive to Gem, the two cell lines were therefore used in subsequent experiments.

### Transfection

Cell culture medium (2 mL) containing 2 × 10^5^ MCF7 and HCC1806 cells was added to each well of a 6-well plate and incubated at 37 °C till 40–60% confluence. Next, A and B transfection solution were prepared. For A solution, 20 pmol Scramble, mimics, inhibitor, and 20 μg NC, siMTDH, and MTDH (Shanghai GenePharma Co., Ltd., China) were dissolved in 50 μL Dulbecco’s modified eagle medium (DMEM, Hyclone, USA). For B solution, 1 μL Lipofectamine 2000 (Invitrogen, USA) was dissolved in 50 μL DMEM. Then, the transfection solution A and solution B were mixed and allowed to stand for 5 min, and then added to the cell culture medium at 37 °C for 24 h.

### Luciferase activity assay

The 3′UTR of MTDH sequence of the site binding to miR-9-3p was inserted into a pmirGLO dual-luciferase vector with *Xho*I and *Xba*I double digestion to construct the generative wildtype (WT) MTDH. The Site-Directed Mutagenesis Kit was used to synthesize the mutant (MUT) MTDH sequence and then inserted into the pmirGLO dual-luciferase vector with *Xho*I and *Xba*I double. Next, the pmirGLO vector containing WT or MUT MTDH sequence was respectively co-transfected with miR-9-3p mimic into HEK293T cells using Lipofectamine 2000 (Invitrogen, USA). After incubation for 48 h, a dual-luciferase reporter gene detection kit (RG088S, Beyotime Biotechnology, China) was used to measure the relative luciferase activity of the cells.

### Cell viability detection

The cells were inoculated into the 96-well plate. 10 μL CCK-8 (96992-100TESTS-F, Sigma-Aldrich, China) solution was added to each well and incubated with the cells in the 96-well plate for 4 h. The absorbance at 450 nm was determined by a microplate reader (BMG Labtech, Germany). The experiment was set up in triplicate for statistical significance.

### Colony formation assay

The cells were transfected, digested, and cultured in 12-well plates at 100 cells per well at 37 °C in a 5% CO_2_ atmosphere for 3 weeks and then the clone number was counted. The conditioned medium was changed every 3 days (d) to observe the formation of clones. The reaction was terminated when the cloned cells were within 50–150 fields, after discarding the medium, the cells were rinsed twice in Dulbecco’s Phosphate Buffered Saline (DPBS, D8662, Sigma-Aldrich, USA). Subsequently, the cells were fixed by 1 mL methanol (34860, Sigma-Aldrich, USA) for 15 min and stained by 1 mL Giemsa (999D715, ThermoFisher Scientific, USA) for 30 min. Colony formation rate = (cells colonies number/seeded cells number) × 100%.

### Cell apoptosis

The transfected cells (1×10^6^/mL) were resuspended in the solution containing 1× Annexin binding buffer (A21009-100ml, Alpha Applied Bioscience, USA), 5 μL fluorescein isothiocyanate (FITC) Annexin V (KGA108-1, KeyGEN BioTECH, http://www.keygentec.com.cn/index.html, China), and 1 μL 100 μg/mL Propidium Iodide (C0080, Beijing Suolaibao Technology Co., Ltd., http://www.solarbio.com/search.php, China). 300 μL 1× Annexin binding buffer was then added into the cell suspension for 15 min after the reaction at room temperature. Finally, FACSCalibur flow cytometry was performed to detect the stained cells (version 10.0, FlowJo, FACS CaliburTM, BD, Franklin Lakes, NJ, USA).

### Wound scratch

The transfected cells were diluted to 1 × 10^6^/mL and then transferred into a petri dish. After the cells were attached to the wall, they were scratched and further cultured. The cells were photographed at 0 h when the scratch operation was completed. 48 h after the cell culture, the migration distance was calculated by Image J software version 1.8.0. Relative migration = (0 h scratch width − 48 h scratch width)/0 h scratch width × 100%.

### Transwell

Transwell assay was performed to detect cell invasion. The upper chamber surface of the transwell chamber bottom membrane was coated by Matrigel (354234, Corning Life Sciences, NY, USA). After cell transfection for 24 h, the cell density was diluted to 1 × 10^6^/mL. The cell suspension was then added to the upper chamber of Transwell (Corning, USA), while the lower chamber was filled with a 10% FBS-containing medium. After the cells were cultured at a chamber at 37 °C for 24 h, the cells in the bottom of the upper chamber were stained with 0.5% crystal violet. The invaded cells were counted at ×100 magnification.

### Tube formation assay

Matrigel matrix gel (250 μL/well, 354433, Becton Dickinson, USA) was added into a 24-well plate to culture the cells for 60 min. The cells were digested by 1 mL EDTA with trypsin (25300054, ThermoFisher Scientific, USA), and the reaction was terminated by adding a serum-containing medium. The HUVECs (American Type Culture Collection) at the logarithmic growth phase (3 × 10^4^ cell/well) were seeded in 96-well plates containing 50 μL matrix gel, and then the densities of MCF7 and HCC1806 cells after the transfection were adjusted to 2 × 10^5^/mL. Subsequently, 500 μL transfected MCF7 and HCC1806 cell supernatants were respectively added into HUVECs and cultured at 37 °C with 5% CO_2_ for 4 h. The number of cell angiogenesis and the tube length were examined under a microscope (Olympus, Japan) from 5 random fields.

### Ethics statement

The animal experiments in this study were approved by the Animal Ethics Committee of Women’s Hospital School of Medicine Zhejiang University, and were conducted in accordance with the guidelines of the National Institutes of Health (USA) for animal experiments.

### Animal model establishment and treatment

A total of forty 4-weeks-old male BALB/c mice were obtained from (CAVENS, Changzhou, China). The mice were raised in an environment at 20–26 °C in 40–70% humidity under a 12 light/12 dark cycle and provided with a normal diet. The mice were divided into 8 groups, namely, NC group, NC + Gem 5 group, MTDH group, MTDH + Gem 5 group, siNC, siNC + Gem 5, siMTDH, siMTDH + Gem 5, with 5 mice in each group.

For establishing a breast cancer model in mice, 2 mL (0.2 × 10^7^ cells) MCF7 cells suspension were subcutaneously injected into each mouse via the armpit of the right forelimb. Three to four days after the subcutaneous inoculation, tumors with a diameter of 4–5 mm could be touched under the armpit of mice, which indicated a successful inoculation. The mice in NC group and NC + Gem 5 group were injected with 2 mL (0.2 × 10^7^ cells) MTDH-NC-transfected MCF7 cell suspension, while those in the MTDH group and MTDH + Gem 5 group were injected with 2 mL (0.2 × 10^7^ cells) MTDH-transfected MCF7 cell suspension. The mice in NC + Gem 5 group and MTDH + Gem 5 group were treated by 5 mg/kg Gem, while those in siNC group and siNC + Gem 5 group were injected with 2 mL (0.2 × 10^7^ cells) MTDH-siNC-transfected MCF7 cell suspension. In addition, the mice in the siMTDH group and siMTDH + Gem 5 group were injected with 2 mL (0.2 × 10^7^ cells) siMTDH-transfected MCF7 cell with transfected MCF7 cell suspension, and those in siNC + Gem 5 group and siMTDH+Gem 5 group were treated by 5 mg/kg Gem.

### Immunohistochemical staining (IHC)

After the collection of tumor tissues from the mice, the tissues were fixed by 4% formaldehyde (SB8430, Solarbio, China) and dehydrated by alcohol. Followed by dewaxing and hydration, the tissues were soaked in citrate buffer solution (pH 6.0) and heated in an 850 W power microwave oven for 10 min for antigen repair. Next, the tissues were washed by distilled water for 2 min, soaked in 3% H_2_O_2_ at room temperature for 10 min to eliminate the activity of endogenous peroxidase. The anti-MTDH antibody (1:100, ab45338, Abcam, USA), anti-p53 (1:100, ab32389, Abcam, USA), and anti-VEGF antibody (1:100, ab32152, Abcam, USA) were added to the tissues and incubated together at 4 °C overnight. Then the tissues were incubated by goat anti-rabbit IgG H&L (HRP) (1:1000; ab205718, Abcam, USA) at 37 °C for 30 min, stained by DAB Horseradish Peroxidase Color Development Kit (Beyotime, Shanghai, China) and observed under an optical microscope (CKX31, Olympus, Japan). When evaluating the results, all researchers were not informed of the assignment of the experimental group.

### Quantitative reverse transcription-polymerase chain reaction (qRT-PCR)

Total RNAs were extracted from the tissues or cells by Trizol reagent (12183555, ThermoFisher Scientific, USA), and NanoDrop™ One/OneC micro-uv-visible spectrophotometer (ND-ONEC-W, Thermo Scientific™, USA) was used to measure RNA concentration (RNA used at 500 ng/μL). Then, the RNAs were reverse-transcribed into cDNAs using the PrimeScript RT kit (RR037A, Takara, China). The mRNA expressions were quantitated by SYBR Green PCR Master Mix (4309155, ThermoFisher Scientific, USA), and the miRNA expressions were determined using a SuperScript™ III Platinum™ SYBR™ Green One-Step qRT-PCR Kit (11736059, ThermoFisher Scientific, USA). GAPDH served as a reference. PCR amplification system consisted of 1 μL distilled water, 3 μL cDNA, 5 μL DNA polymerase, and 1 μL Primer. The ABI7500 system (Applied Biosystems) was used for the qRT-PCR reaction. Changes in relative mRNA expressions were calculated by 2^−ΔΔCT^ method [14]. The PCR cycle system was set as follows: at 95 °C for 10 min, at 95 °C for 15 s, at 72 °C for 15 s, for a total of 40 cycles. All the primer sequences of qRT-PCR used in this study were listed in Supplementary Table S1.

### Western blot

RIPA buffer (P0013B, Beyotime Biotechnology, China) was used to lyse the total proteins in tissues and cells, and protein concentration was measured by BCA kit (P0012S, Beyotime Biotechnology, China). The proteins were separated by electrophoresis on 15% dodecyl sulfate sodium salt-polyacrylamide gel (P0508S, Beyotime Biotechnology, China) and moved to polyvinylidene fluoride membranes (FFP24, Beyotime Biotechnology, China), which were then sealed by 5% milk at 25 °C for 1 h. Primary antibodies (listed in Supplementary Table S2) were incubated with the protein at 4 °C overnight. The membranes incubated with the primary antibody were then incubated with the secondary antibody (Protein tech, USA) for 2 h, and washed by PBS three times. ECL kit (D3308, Beyotime Biotechnology, China) was applied to develop the protein bands, followed by scanning with a super-sensitive multifunctional imager (Amersham Imager 600, General Electric Company, USA).

### Statistical analysis

The data were analyzed by SPSS 18.0 (Chicago, USA) and shown as mean ± standard deviation. Group differences were measured by ANOVA, and a *t*-test was used to compare the data difference between the two groups. Each treatment was carried out in triplicate. *P* < 0.05 was defined as statistically significant.

## Results

### Bioinformatics analysis of MTDH expression and prognosis of the breast cancer patients

TCGA data analysis showed that MTDH was high-expressed in the breast cancer tissues and this was related to a poor prognosis (Supplementary Fig S1A). The fragment data of all samples was analyzed by GISTIC 2.0. The results showed that the copy number of MTDH increased significantly (Supplementary Fig S1B).

### Expression of MTDH in tissues and cells of breast cancer patients

**Fig. 1:**
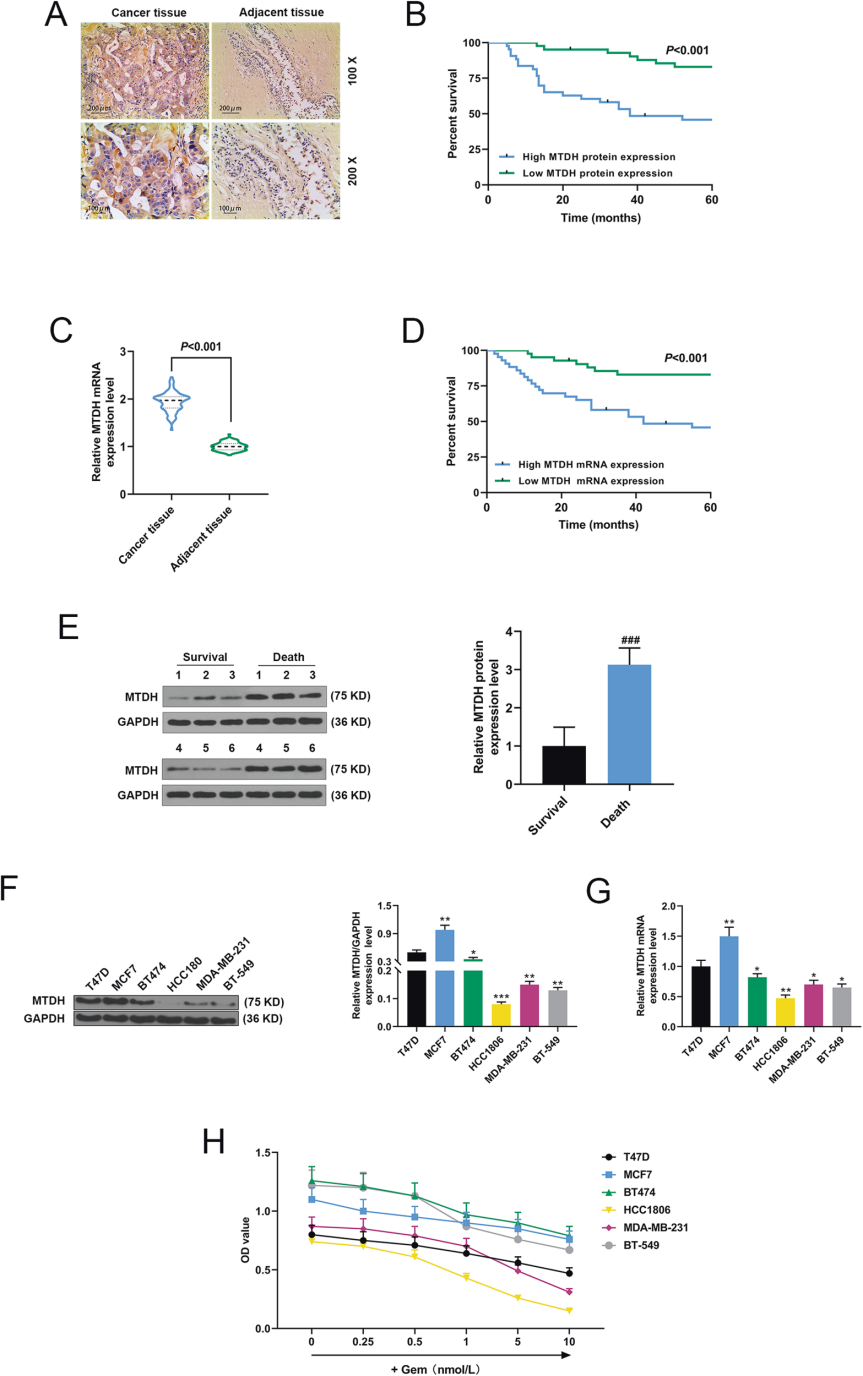
Expression of MTDH in tissues and cells of breast cancer patients. **A** Immunohistochemistry was performed to detect MTDH expression in cancer tissues and adjacent tissues of breast cancer patients. **B** According to the expression of MTDH detected by immunohistochemistry in cancer tissues, Kaplan–Meier survival analysis was used to analyze the relationship between the survival rate of breast cancer patients and the expression of MTDH. **C** QRT-PCR was performed to detect MTDH expression in cancer tissues and adjacent tissues of breast cancer patients. **D** The survival of breast cancer patients was analyzed according to the expression of MTDH in cancer tissues by Kaplan–Meier survival analysis. **E** The protein expression of MTDH in patients who died and surviving patients during follow-up was measured by Western blotting. **F** Expression of MTDH in human breast cancer cell lines (T47D, MCF7, BT474, HCC1806, MDA-MB-231, and BT-549) was detected by Western blot (*n* = 3, *P < 0.05, **P < 0.01, ***P < 0.001, vs. T47D). **G** Expression of MTDH in human breast cancer cell lines (T47D, MCF7, BT474, HCC1806, MDA-MB-231 and BT-549) was detected by qRT-PCR (*n* = 3, *P < 0.05, **P < 0.01, ***P < 0.001, vs^.^ T47D). **H** MTT assay was used to detect cellular viability treated by different concentrations of gemcitabine (Gem). MTDH Metadherin, QRT-PCR quantitative real-time polymerase chain reaction.

Immunohistochemical staining was applied to detect MTDH expression in tissues of breast cancer patients and adjacent tissues, and we found that MTDH expression in cancer tissues was significantly higher than that in adjacent tissues (Fig. 1A). Next, as shown in Fig. 1B, the relationship between patient survival rate and MTDH protein expression was analyzed by Kaplan–Meier survival analysis, and we found that patients with higher protein expression of MTDH developed poorer prognosis, while those with lower protein expression of MTDH showed a more satisfactory prognosis. Furthermore, qRT-PCR results demonstrated that MTDH mRNA expression in cancer tissues was noticeably higher than that in adjacent tissues (Fig. 1C, *P* < 0.001). The median mRNA expression of MTDH in cancer tissues served as the standard, and patients with MTDH expression higher than the median in their cancer tissues were assigned into the MTDH high-expression group, while those with the expression lower than the median were in the MTDH low-expression group. We conducted a survival analysis to determine the expression level of MTDH in the cancer tissues, and observed that patients with higher expression of MTDH developed poorer prognosis, while those with lower expression of MTDH had a more satisfactory prognosis (*P* < 0.001, Fig. 1D). Moreover, the protein expression of MTDH in both who died and survived during follow-up was measured by Western blotting, and the data demonstrated that the expression of MTDH in patients who died during follow-up was significantly higher than that in survived patients (*P* < 0.001, Fig. 1E). In addition, the expression of MTDH in T47D, BT474, HCC1806, MDA-MB-231, and BT-549 cells were determined by Western blot. As shown in Fig. 1F, MTDH expression was significantly increased in MCF7 cells, but was greatly decreased in HCC1806 cells compared with T47D cells (*P* < 0.01, *P* < 0.05, *P* < 0.001). Moreover, qRT-PCR showed similar results in the detection of MTDH expression in the cells (*P* < 0.01, *P* < 0.05, Fig. 1G).

### The effects of Gem on the biological characteristics of breast cancer cells and protein expression in the cells

CCK-8 was performed to detect the sensitivity of the cells to Gem after the cells were treated by Gem at different concentrations (0.25, 0.5, 1, 5, 10 nM) for 72 h. We observed that MCF7 showed the lowest sensitivity to Gem, while HCC1806 was the most sensitive to Gem, indicating that higher MTDH expression in breast cancer cells was correlated with a lower sensitivity of cells to Gem (Fig. 1H). Flow cytometry was carried out to determine the effect of Gem on apoptosis of MCF7 cell and HCC1806 cells, as shown in Supplementary Fig. S2A, when compared with MCF7, Gem obviously treated HCC1806 apoptosis (*P* < 0.05). Subsequently, the effect of Gem on cleaved caspase-3 in breast cancer cells was examined by Western blot, and it could be observed that Gem increased expression of cleaved caspase-3 in HCC1806 cells compared with MCF7 cells (*P* < 0.05, Supplementary Fig. S2B). Furthermore, the colony formation assay was performed to detect the effect of Gem on the growth of MCF7 cells and HCC1806 cells, and we found that Gem significantly decreased the clones of HCC1806 cells when compared with MCF7 cells (*P* < 0.05, *P* < 0.001, Supplementary Fig. S2C). P53 is a tumor suppressor gene regulating the cell cycle and preventing cells from becoming cancerous [15, 16]. VEGF can specifically stimulate the proliferation of endothelial cells, therefore is closely related to pathological angiogenesis [17]. VEGF expression is abnormally increased in the serum and tumor of cancer patients[18]. Therefore, we detected the expressions of p53 and VEGF in Gem-treated cells by Western blot (Supplementary Fig. S2D) and qRT-PCR (Supplementary Fig. S2E). As shown in Fig. 3D, E, the data demonstrated that compared with MCF7 conditioned medium, the expression of p53 was significantly increased, and VEGF expression was inhibited in Gem-treated HCC1806 cells conditioned medium (*P* < 0.05, *P* < 0.001).

### The effects of MTDH on the biological characteristics of Gem-treated breast cancer cells

**Fig. 2:**
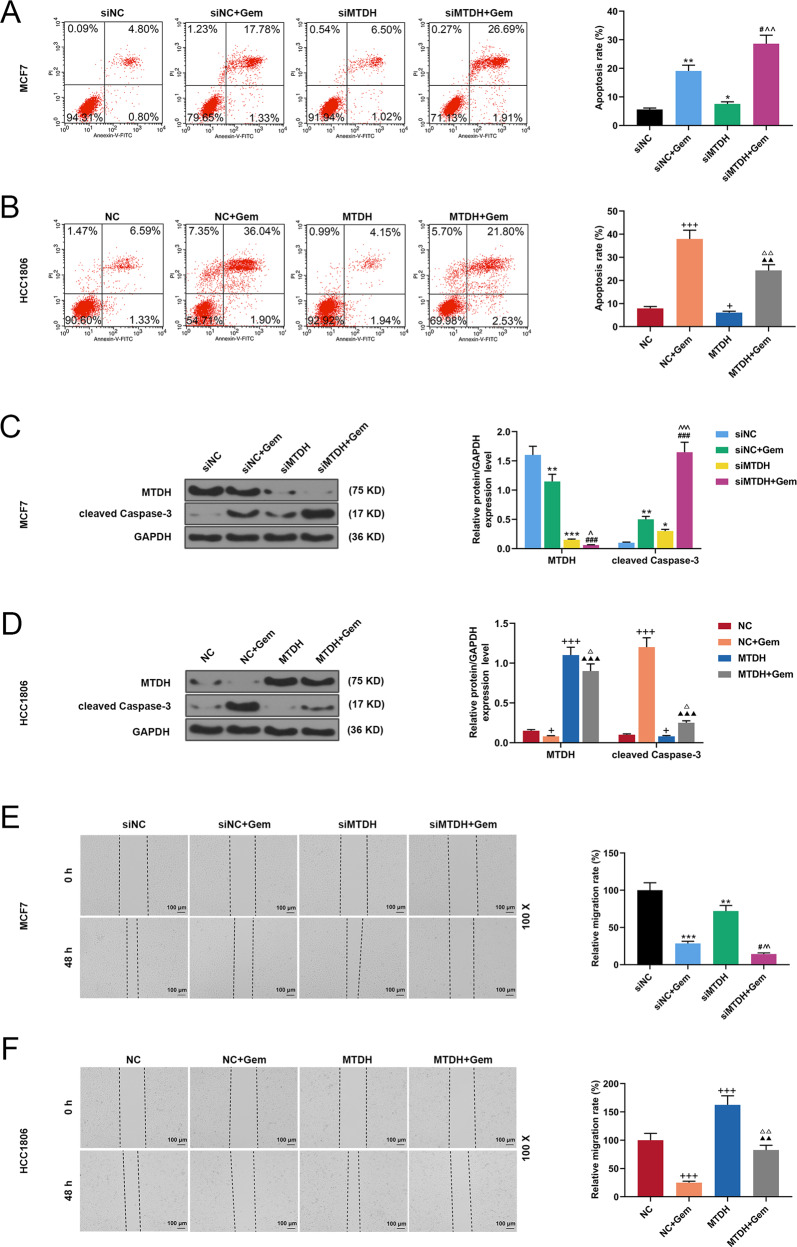
The effects of MTDH on apoptosis and migration of Gem-treated breast cancer cells. **A** Flow cytometry was used to determine the effect of silencing MTDH expression on apoptosis of MCF7 cells treated by Gem (*n* = 3, *P < 0.05, **P < 0.01, vs. siNC; ^#^*P* < 0.05, vs. siNC + Gem; ^^^*P* < 0.05, ^^^^*P* < 0.01, vs. siMTDH). **B** Flow cytometry was used to determine the effect of overexpressed MTDH on apoptosis of HCC1806 cells treated by Gem (*n* = 3, ^+^*P* < 0.05, ^+++^*P* < 0.001, vs. NC; ^▲▲^*P* < 0.01, vs. NC + Gem; ^△△^*P* < 0.01, vs. MTDH). **C** The expression of cleaved caspase-3 and MTDH were detected by Western blot in MCF7 cells treated by siMTDH and Gem (*n* = 3, *P < 0.05, **P < 0.01, ***P < 0.001, vs. siNC; ^###^*P* < 0.001, vs. siNC + Gem; ^^^*P* < 0.05, ^^^^^*P* < 0.001, vs. siMTDH). **D** The expression of cleaved caspase-3 and MTDH were detected by Western blot in HCC1806 cells treated by MTDH and Gem (*n* = 3, ^+^*P* < 0.05, ^+++^*P* < 0.001, vs. NC; ^▲▲▲^*P* < 0.001, vs. NC + Gem; ^△^*P* < 0.05, vs. MTDH). **E** Wound wound healing assay was used to detect MCF7 cell migration. (*n* = 3, **P < 0.01, ***P < 0^.^001, vs. siNC; ^#^*P* < 0.05, vs. siNC + Gem; ^^^^*P* < 0.01, vs. siMTDH). **F** Wound scratch assay was used to detect HCC1806 cell migration (n = 3, ^+++^*P* < 0.001, vs. NC; ^▲▲^*P* < 0.01, vs. NC + Gem; ^△△^*P* < 0.01, vs. MTDH). MTDH Metadherin, NC negative control.

**Fig. 3:**
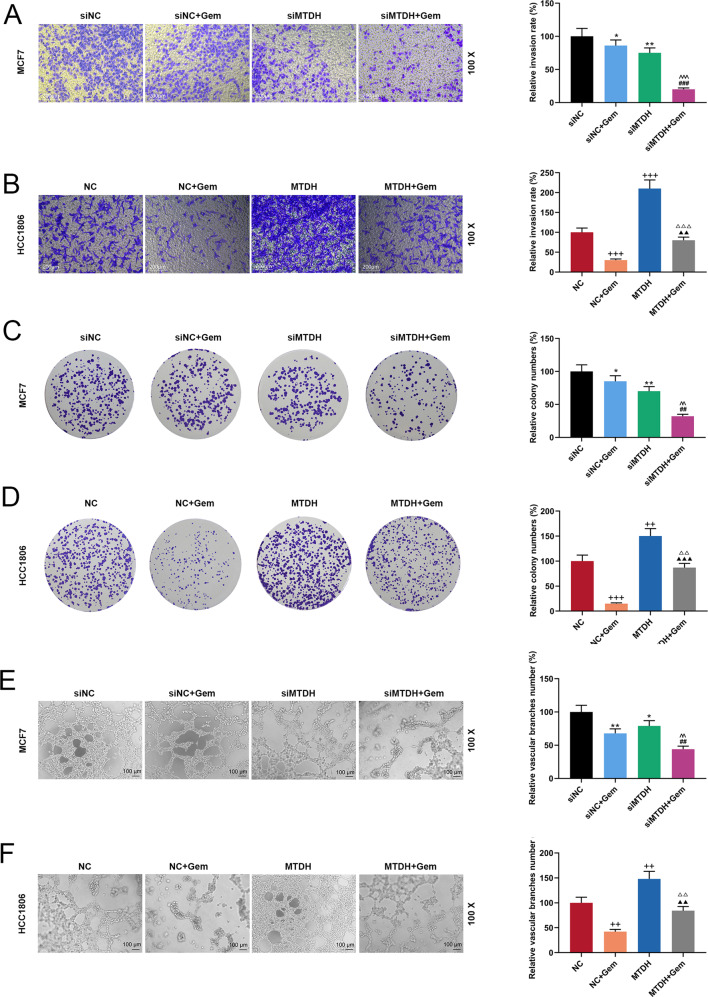
The effects of MTDH on invasion and growth of Gem-treated breast cancer cells. **A** Transwell examined the effects of silenced MTDH on MCF7 cell invasion (*n* = 3, *P < 0.05, **P < 0.01, vs. siNC; ^###^*P* < 0.001, vs. siNC + Gem; ^^^^^*P* < 0.001, vs. siMTDH). **B** Transwell examined the effects of overexpressed MTDH on HCC1806 cell invasion (*n* = 3, ^+++^*P* < 0.001, vs. NC; ^▲▲^*P* < 0.01, vs. NC + Gem; ^△△△^*P* < 0.001, vs. MTDH). **C** The number of MCF7 cell clones was determined by cell cloning experiment (*n* = 3, *P < 0.05, **P < 0.01, vs. siNC; ^##^*P* < 0.01, vs. siNC + Gem; ^^^^*P* < 0.01, vs. siMTDH). **D** The number of HCC1806 cell clones was determined by cell cloning experiment (*n* = 3, ^++^*P* < 0.01, ^+++^*P* < 0.001, vs. NC; ^▲▲▲^*P* < 0.001, vs. NC + Gem; ^△△^*P* < 0.01, vs. MTDH). **E** The effect of silenced MTDH on angiogenesis of MCF7 cells was detected by tube formation assay (*n* = 3, *P < 0.05, **P < 0.01, vs. siNC; ^##^*P* < 0.01, vs. siNC + Gem; ^^^^*P* < 0.01, vs. siMTDH). **F** The effect of overexpressed MTDH on angiogenesis of HCC1806 cells was detected by tube formation assay (*n* = 3, ^++^*P* < 0.01, vs. NC; ^▲▲^*P* < 0.01^,^ vs. NC + Gem; ^△△^*P* < 0.01, vs. MTDH). MTDH Metadherin, NC negative control.

To further investigate the effect of MTDH on Gem-treated breast cancer cells, the MTDH silencing vector and MTDH overexpression vector were respectively transfected into MCF7 cells and HCC1806 cells. Flow cytometry showed that silencing MTDH greatly promoted cell apoptosis of Gem-treated MCF7 (*P* < 0.01, *P* < 0.05, Fig. 2A), while overexpressed MTDH obviously inhibited the apoptosis of Gem-treated HCC1806 cells (*P* < 0.01, *P* < 0.05, *P* < 0.001, Fig. 2B). Subsequently, the effect of MTDH on cleaved caspase-3 in Gem-treatment breast cancer cells was further examined by Western blot, and we found that in Gem-treated MCF7 cells silencing MTDH increased the expression of cleaved caspase-3 (*P* < 0.01, *P* < 0.05, *P* < 0.001, Fig. 2C), which was partially reversed by overexpressed MTDH (*P* < 0.01, *P* < 0.05, *P* < 0.001, Fig. 2D). Furthermore, the effect of MTDH on the migration of Gem-treatment breast cancer cells was further determined by performing a wound-healing assay. It has been found that silencing MTDH inhibited MCF7 cell migration and enhanced the inhibitory effect of Gem on MCF7 cell migration (*P* < 0.01, *P* < 0.05, *P* < 0.001, Fig. 2E). Moreover, overexpressed MTDH promoted migration and attenuated Gem-treated inhibition of cell migration (*P* < 0.05, *P* < 0.001, Fig. 2F). Transwell was performed to detect the effect of MTDH on the invasion of Gem-treated breast cancer cells, and the data indicated that silencing MTDH inhibited the cell invasion but significantly promoted the inhibition of Gem-treated cell invasion (*P* < 0.01, *P* < 0.05, *P* < 0.001, Fig. 3A), whereas overexpression of MTDH enhanced cell invasion and significantly reduced cell invasion previously inhibited by Gem (*P* < 0.05, *P* < 0.001, Fig. 3B). Colony formation assay was conducted to detect the effect of MTDH on Gem-treatment breast cancer cell clone formation, and the results showed that silencing MTDH inhibited clone formation and significantly enhanced the inhibitory effect of Gem-treated clone formation of MCF7 cells (*P* < 0.01, *P* < 0.05, Fig. 3C). However, overexpressed MTDH enhanced clone formation and noticeably reduced the inhibition of clone formation of HCC1806 cells treated by Gem (*P* < 0.05, *P* < 0.001, Fig. 3D). Silencing MTDH inhibited angiogenesis and significantly promoted the inhibitory effect of Gem-treated angiogenesis of MCF7 cells conditioned medium (*P* < 0.01, *P* < 0.05, Fig. 3E), which were all greatly reversed by overexpressed MTDH (*P* < 0.05, Fig. 3F).

### The effects of MTDH on the protein expressions of Gem-treated breast cancer cells

**Fig. 4:**
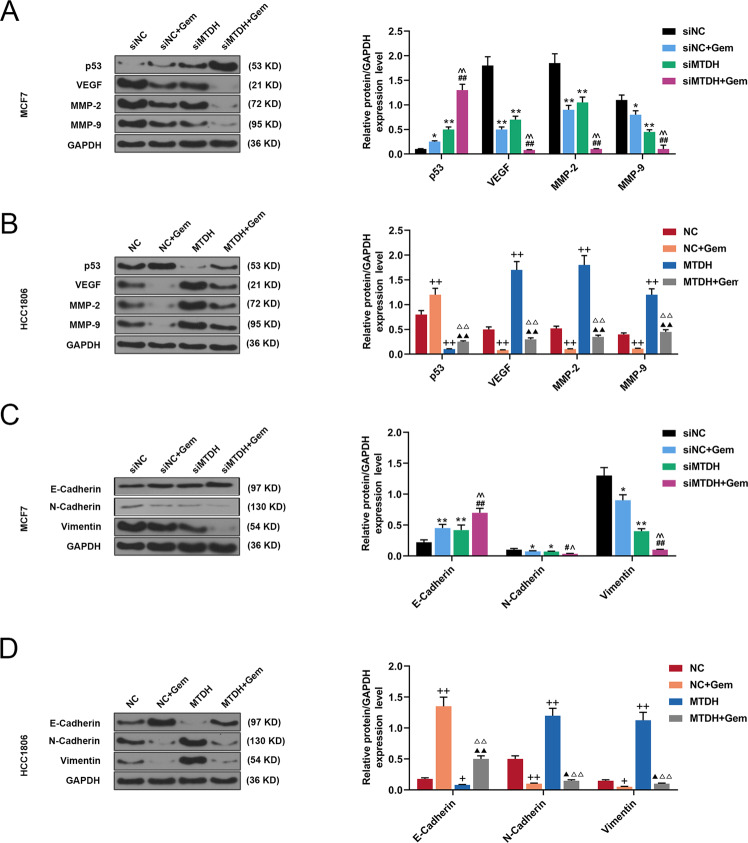
The effects of MTDH on protein expressions of Gem-treated breast cancer cells. **A** The effects of silenced MTDH on expressions of p53, VEGF, MMP-2, and MMP-9 in MCF7 cells were detected by Western blot (*n* = 3, *P < 0.05, **P < 0.01, vs. siNC; ^##^*P* < 0.01, vs. siNC + Gem; ^^^^*P* < 0.01, vs. siMTDH). **B** The effects of overexpressed MTDH on expressions of p53, VEGF, MMP-2, and MMP-9 in HCC1806 cells were detected by Western blot (*n* = 3, ^++^*P* < 0.01, vs. NC; ^▲▲^*P* < 0.01, vs. NC + Gem; ^△△^*P* < 0.01, vs. MTDH). **C** The effects of silenced MTDH on expressions of E-Cadherin, N-Cadherin, and Vimentin in MCF7 cells were detected by Western blot (*n* = 3, *P < 0.05, **P < 0.01, vs. siNC; ^##^*P* < 0.01, vs. siNC + Gem; ^^^^*P* < 0.01, vs. siMTDH). **D** The effects of overexpressed MTDH on expressions of E-Cadherin, N-Cadherin, and Vimentin in HCC1806 cells were detected by Western blot (*n* = 3, ^+^*P* < 0.05, ^++^*P* < 0.01, vs. NC; ^▲^*P* < 0.05, ^▲▲^*P* < 0.01, vs. NC + Gem; ^△△^*P* < 0.01, vs. MTDH). VEGF vascular endothelial growth factor, MTDH Metadherin, NC negative control.

Matrix metalloproteinases (MMPs) are closely related to tumor invasion and metastasis [19, 20]. Therefore, in this study, we also detected the effect of MTDH on Gem-treated breast cancer cells and the expressions of MMP-2 and MMP-9 proteins. The related data showed that in Gem-treated MCF7 cells, silencing MTDH increased expression of p53 but inhibited expressions of VEGF, MMP-2, and MMP-9 (*P* < 0.01, *P* < 0.05, Fig. 4A). However, overexpression of MTDH in HCC1806 cells produced the opposite effects on the expressions of p53, VEGF, MMP-2, and MMP-9 (*P* < 0.05, Fig. 4B). Epithelial–mesenchymal transition (EMT) refers to the process during which epithelial cells lose polarity and epithelial cell characteristics and obtain interstitial phenotype and invasive potential. EMT plays a key role in tumor invasion and metastasis [21, 22] and is mainly characterized by the transformation of cell morphology from epithelial to interstitial. The decreased expressions of epithelial cell markers (such as E-cadherin) and increased expressions of stromal cell markers (such as N-Cadherin and Vimentin) are indicative of EMT [23, 24]. Thus, the current study also detected the effects of MTDH on the expressions of E-Cadherin, N-Cadherin, and Vimentin in Gem-treated breast cancer cells, and found that silencing MTDH promoted expression of E-Cadherin and inhibited expressions of N-Cadherin and Vimentin in Gem-treated MCF7 cells (*P* < 0.01, *P* < 0.05, Fig. 4C). Overexpression of MTDH in HCC1806 cells had the opposite effects on the expressions of E-Cadherin, N-Cadherin, and Vimentin (*P* < 0.01, *P* < 0.05, Fig. 4D).

### The effects of MTDH on tumor growth and protein expression in Gem-treated tumor in mice

**Fig. 5:**
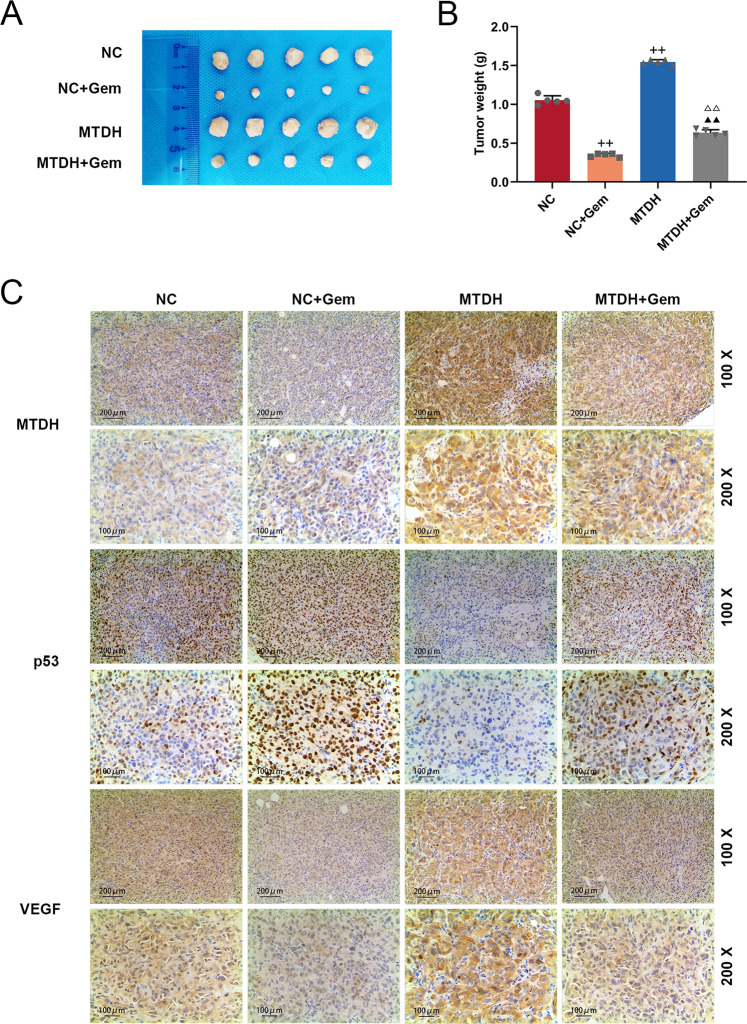
The effects of MTDH on the tumor growth and protein expression in Gem-treated tumor mice. Mouse tumor-bearing model was established and the mice were treated by MTDH and Gem, the size (**A**) and weight (**B**) of tumors were measured (*n* = 3, ^++^*P* < 0.01, vs. NC; ^▲▲^*P* < 0.01, vs^.^ NC + Gem; ^△△^*P* < 0.01, vs. MTDH). **C** Immunohistochemistry was used to detect the expressions of MTDH, p53, and VEGF in tumor tissues. VEGF vascular endothelial growth factor, MTDH Metadherin, NC negative control.

The effects of MTDH and Gem on breast cancer tumors in vivo were examined by establishing a tumor model in mice. The results showed that silencing MTDH inhibited tumor size, promoted Gem on inhibiting tumor size (Supplementary Fig. S3A), and reduced tumor weight of the model mice (Supplementary Fig. S3B). The effects of Gem and siMTDH on protein expressions in tumor tissues were evaluated by performing immunohistochemical staining, and we found that Gem and silencing MTDH promoted the expression of p53 and inhibited the expressions of MTDH and VEGF. Moreover, silencing MTDH enhanced the effects of gem on the protein expressions in tumors of the model mice (Supplementary Fig. S3C). In addition, overexpressed MTDH increased tumor size and reversed the inhibitory effect of Gem on tumor size of the model mice (Fig. 5A), and overexpressed MTDH increased tumor weight and reversed the inhibitory effect of Gem on tumor weight of the mice (Fig. 5B). The effects of Gem and MTDH on the protein expressions in tumor tissues were detected by performing immunohistochemical staining. The data demonstrated that overexpression of MTDH inhibited p53 expression, increased the expressions of MTDH and VEGF, and partially reversed the effects of gem on protein expressions (Fig. 5C).

### MiR-9-3p bound to MTDH and affected the viability, migration, and proliferation of breast cancer cells

**Fig. 6:**
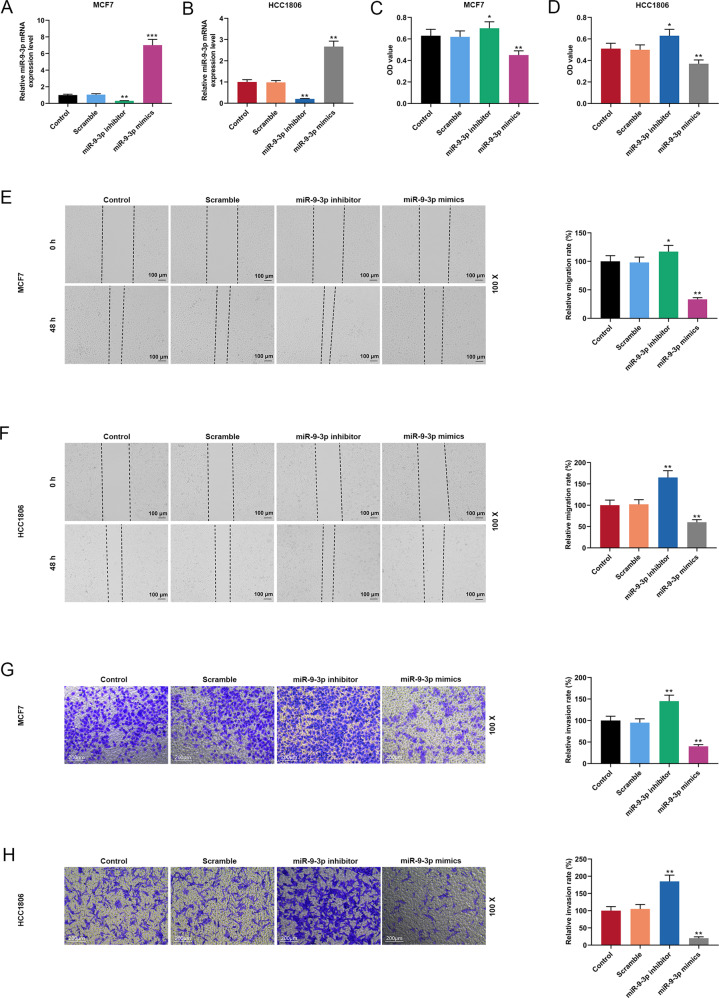
MiR-9-3p affects the viability, migration, and proliferation of breast cancer cells. **A** QRT-PCR was used to detect the expression of miR-9-3p in MCF7 cell transfected with miR-9-3p inhibitor and mimics. **B** QRT-PCR was used to detect the expression of miR-9-3p in HCC1806 cells transfected with miR-9-3p inhibitor and mimics. **C** The viability of MCF7 cells was measured by CCK-8. **D** The viability of HCC1806 cells was measured by CCK-8. **E** Wound scratch assay was used to test the migration ability of transfected miR-9-3p inhibitor and mimics MCF7 cells. **F** Wound scratch assay was used to determine the migration ability of transfected miR-9-3p inhibitor and mimics HCC1806 cells. **G** Transwell was used to examine the effects of miR-9-3p inhibitor and mimics on MCF7 cell invasion. **H** Transwell examined the effects of miR-9-3p inhibitor and mimics on HCC1806 cell invasion. (*n* = 3, **P < 0.001, vs. Control). QRT-PCR quantitative real-time polymerase chain reaction, CCK-8 cell counting kit-8.

MiRNAs play important roles in regulating gene expressions as well as in the treatments of cancer and many other diseases [25, 26]. Therefore, the miRNA targeting MTDH was predicted and analyzed by Targetscan and miRDB websites. As shown in the Supplementary Fig. S4A, TargetScan and miRDB websites predicted a total of 29 miRNAs targeting MTDH, and the following potential target miRNAs for MTDH were further determined by literature review: miR-193b, miR-26b-5p, miR-26a-5p, let-7g, let-7a, let-7b, miR-128, miR-493, and miR-9-3p. Then qRT-PCR was performed to detect the expressions of miR-193b, miR-26b-5p, miR-26a-5p, let-7g, let-7a, let-7b, miR-128, miR-493, and miR-9-3p in MCF7 and HCC1806 cells treated by Gem. We found that miR-9-3p was significantly elevated in both Gem-treated MCF7 and HCC1806 cells (*P* < 0.001, Supplementary Fig. S4B, C), suggesting that it might be involved in the inhibition of cancer cell growth and migration under Gem treatment. Target binding sites of miR-9-3p and MTDH were predicted by TargetScan (Supplementary Fig. S4D), and we constructed pmirGLO dual-luciferase reporter vectors containing MTDH-WT and MTDH-MUT and co-transfected then with miR-9-3p mimic into HEK293T cells. The luciferase viability of MTDH-WT was significantly reduced (*P* < 0.001, Supplementary Fig. S4E). The effect of miR-9-3p on the biological characteristics of breast cancer cells was confirmed by further conducting transfection experiments. We observed that the expression of miR-9-3p was significantly inhibited in MCF7 cells transfected with miR-9-3p inhibitor but was greatly increased in MCF7 cells transfected with miR-9-3p mimics (*P* < 0.05, *P* < 0.001, Fig. 6A). Similarly, the expression of miR-9-3p was sharply inhibited in HCC1806 cells transfected with miR-9-3p inhibitor and was significantly increased in HCC1806 cells transfected with miR-9-3p mimics (*P* < 0.05, Fig. 6B). CCK-8 assay results showed that inhibition of miR-9-3p expression promoted MCF7 cell viability, which was inhibited by overexpression of miR-9-3p (*P* < 0.01, *P* < 0.05, Fig. 6C). Moreover, inhibiting miR-9-3p expression promoted HCC1806 cell viability, which was inhibited by overexpression of miR-9-3p (*P* < 0.01, *P* < 0.05, Fig. 6D). In addition, wound scratch was performed to detect the effect of miR-9-3p on the migration of breast cancer cells, and we found that inhibition of miR-9-3p expression promoted MCF7 cell migration, which was inhibited by overexpression of miR-9-3p (*P* < 0.01, *P* < 0.05, Fig. 6E). Moreover, inhibition of miR-9-3p expression promoted HCC1806 cell migration, which was inhibited by overexpression of miR-9-3p (*P* < 0.05, Fig. 6F). Transwell was performed to detect the effects of miR-9-3p on the invasion of breast cancer cells, and we observed that inhibition of miR-9-3p expression promoted MCF7 cell invasion, which was inhibited by overexpression of miR-9-3p (*P* < 0.01, *P* < 0.05, Fig. 6G). Transwell results showed that inhibition of miR-9-3p expression promoted HCC1806 cell invasion, while was inhibited by overexpression of miR-9-3p (*P* < 0.05, Fig. 6H).

### The effects of miR-9-3p on the biological characteristics and apoptotic protein expressions of Gem-treated breast cancer cells through targeting MTDH

**Fig. 7:**
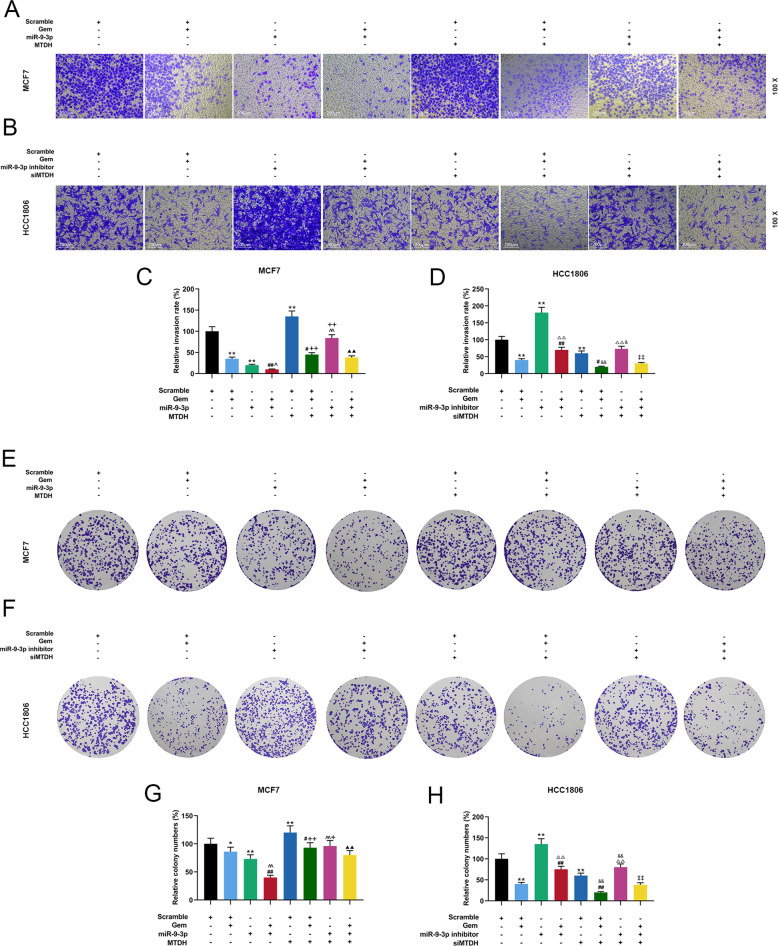
The effects of miR-9-3p on invasion and growth of Gem-treated breast cancer cells by targeting MTDH. **A**, **C** Transwell was used to determine the effects of overexpressed MTDH, miR-9-3p mimics, and Gem on the invasion of MCF7 cells. **B**, **D** Transwell was used to determine the effects of siMTDH, miR-9-3p inhibitor, and Gem on the invasion of HCC1806 cells. **E**, **G** Cell cloning experiment was used to detect the effects of overexpressed MTDH, miR-9-3p mimics and Gem on clone ability of MCF7 cells. **F**, **H** Cell cloning experiment was used to detect the effects of siMTDH, miR-9-3p inhibitor, and Gem on clone ability of HCC1806 cells. (*n* = 3, *P < 0.05, **P < 0.01, ***P < 0.001, vs. Scramble; ^##^*P* < 0.01, vs. Scramble + Gem; ^^^^*P* < 0.01, vs. miR-9 mimics) (*n* = 3, ^+^*P* < 0.05, ^++^*P* < 0.01, vs. Scramble + MTDH; ^▲^*P* < 0.05, ^▲▲^*P* < 0^.^01, vs. miR-9 mimics + Gem; ^△△^*P* < 0.01, vs. miR-9 inhibitor; ^&&^*P* < 0.01, vs. Scramble + siMTDH; ^‡‡^*P* < 0.01, vs. miR-9 inhibitor + Gem). MTDH Metadherin.

**Fig. 8:**
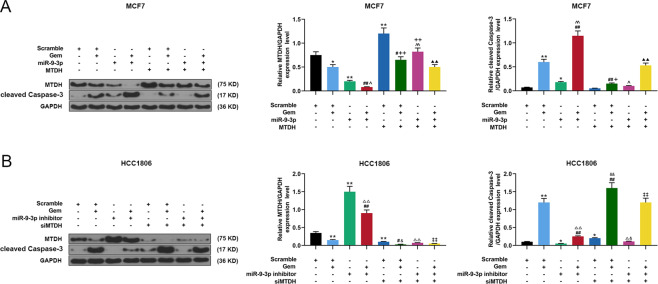
The effects of miR-9-3p on apoptotic protein expressions of Gem-treated breast cancer cells by targeting MTDH. **A** Western blot and qRT-PCR were used to determine the effect of overexpressed MTDH, miR-9-3p mimics, and Gem on the expression of MTDH and cleaved caspase-3 in MCF7 cells. **B** Western blot and qRT-PCR were used to determine the effects of siMTDH, miR-9-3p inhibitor, and Gem on expressions of MTDH and cleaved caspase-3 of HCC1806 cells. (*n* = 3, *P < 0.05, **P < 0.01, ***P < 0.001, vs. Scramble; ^##^*P* < 0.01, vs. Scramble + Gem; ^^^^*P* < 0.01, vs. miR-9 mimics) (*n* = 3, ^+^*P* < 0.05, ^++^*P* < 0.01, vs. Scramble + MTDH; ^▲^*P* < 0.05, ^▲▲^*P* < 0.01, vs. miR-9 mimics + Gem; ^△△^*P* < 0.01, vs. miR-9 inhibitor; ^&&^*P* < 0.01, vs. Scramble + siMTDH; ^‡‡^*P* < 0.01, vs. miR-9 inhibitor + Gem). MTDH Metadherin, QRT-PCR quantitative real-time polymerase chain reaction.

We further investigated the effects of MTDH targeting miR-9-3p on the biological characteristics and apoptotic protein expressions of Gem-treated breast cancer cells. The results of flow cytometry showed that overexpression of miR-9-3p significantly promoted the apoptosis of Gem-treated MCF7 cells, while overexpression of MTDH partially reversed such an effect (*P* < 0.05, Supplementary Fig. S5A, C). Similarly, inhibiting miR-9-3p significantly suppressed the apoptosis of Gem-treated HCC1806 cells, while silencing MTDH partially reversed the effect (*P* < 0.05, Supplementary Fig. S5B, D). Wound scratch assay demonstrated that overexpression of miR-9-3p inhibited migration and enhanced Gem-treated migration inhibition, while overexpression of MTDH partially reversed the effect (*P* < 0.05, Supplementary Fig. S5E, G). Similarly, Silencing miR-9-3p promoted migration and attenuated Gem-treated inhibition of migration, which was partially reversed by silencing MTDH (*P* < 0.05, Supplementary Fig. S5F, H). Transwell was performed to detect cell invasion, and the results showed that overexpression of miR-9-3p inhibited cell invasion and significantly promoted Gem-treated inhibition of cell invasion, which was partially reversed by overexpression of MTDH (*P* < 0.05, Fig. 7A, C). Similarly, silencing miR-9-3p enhanced cell invasion and significantly reduced Gem-treated inhibition of cell invasion, while silencing MTDH partially reversed such an effect (*P* < 0.05, Fig. 7B, D). Moreover, colony formation assay was performed to detect the effect of MTDH targeting miR-9-3p on clone formation of Gem-treated breast cancer cells, and we found that overexpression of miR-9-3p inhibited clone formation and significantly promoted Gem-treated clone formation inhibition, which was partially reversed by overexpression of MTDH (*P* < 0.05, Fig. 7E, G). Similarly, silencing miR-9-3p enhanced clone formation and significantly reduced Gem-treated inhibition of clone formation, while silencing MTDH partially reversed such an effect (*P* < 0.05, Fig. 7F, H). In addition, overexpression of miR-9-3p inhibited MTDH expression, promoted cleaved caspase-3 expression, and enhanced the regulation of Gem on the protein expressions. However, overexpression of MTDH partially reversed the effect of miR-9-3p overexpression on regulating protein expressions (*P* < 0.05, Fig. 8A). miR-9-3p promoted MTDH expression and inhibited cleaved caspase-3 expression. Inhibiting miR-9-3p could reverse the regulation of Gem on protein expressions, while inhibiting MTDH partially reversed the effect of low-expressed miR-9-3p on regulating protein expressions (*P* < 0.05, Fig. 8B).

## Discussion

MTDH gene, also known as astrocyte-elevated gene-1, was originally considered as a single transmembrane protein at 8q22 by rapid subtraction hybridization. MTDH is treated by HIV-1 infection of human embryonic astrocytes or by tumor necrosis factor-ɑ [27]. In 2004, Brown and Ruoslahti found that breast cancer cells with high-expressed MTDH transfer easily to the lungs, but its metastasis into the lungs is greatly reduced by knocking out the MTDH gene through siRNA technology in experimental mice [28]. These findings indicated the MTDH gene is involved in mediating metastasis of breast cancer into the lung.

In addition, TCGA data analysis showed that the prognosis of breast cancer patients with high-expressed MTDH is poor. In this study, by examining breast cancer tissues, we found that the expression of MTDH in the cancer tissues was significantly increased. Moreover, the prognosis analysis demonstrated that higher expression of MTDH was related to a worse prognosis, while lower MTDH expression indicated a better prognosis. At the same time, we detected the expression of MTDH in breast cancer cell lines, and found that MTDH was the highest in MCF7 cells but the lowest in HCC1806 cells. After Gem treatment, the cell apoptosis and cloned and formation were detected. In addition, the cleaved caspase-3 gene, which is a tumor suppressor gene, inhibits cancer growth and promotes apoptosis of cancer cells, and its transcription regulates the chemotherapeutic resistance to angiogenesis [29, 30]. VEGF is a growth factor specifically acting on vascular endothelial cells, and plays an important role in the formation of tumor neovascularization [31]. P53 gene is a tumor suppressor gene with the highest mutation frequency in tumors, and cells with deletion or mutation of P53 show resistance to chemotherapy drugs [32]. EMT, which is a major characteristic of invasive tumor cells, is characterized by decreased E-Cadherin expression and increased N-Cadherin expression [33]. Vimentin, a marker of EMT, is also widely expressed in normal mesenchymal cells [34]. Moreover, the activation of MMPs can promote the invasion of tumor cell lines, and it is therefore closely related to the malignant phenotype of tumor cells [35, 36]. In this study, we detected the epigenetic characteristics of the breast cancer cells and the expressions of proteins regulating the apoptosis, invasion of the cells, and expressions of other related proteins. We found that MCF7 cells with high-expressed MTDH had the lowest sensitivity to Gem, while HCC1806 cells with low-expressed MTDH showed the highest sensitivity to Gem, suggesting that higher expression of MTDH in breast cancer cells was negatively related to the sensitivity of cells to Gem. siMTDH and MTDH were transfected into breast cancer cells for in vitro experiments, and we explored the effects of MTDH on the sensitivity of mice to Gem by in vivo mouse tumor formation experiments, as we expected, our in vitro and in vivo experiments confirmed the previous results.

Abnormally expressed miRNAs play important roles in the occurrence and progression of various diseases in humans including in malignant tumors [37]. It has been confirmed that various miRNAs including miR-451, miR-21, miR-328, and miR-222 are involved in regulating the sensitivity of breast tumor cells to chemotherapeutic drugs [38–40]. MiRNAs are cell-specific and can affect drug tolerance in a drug-specific manner. Increased expression of miR-34a is associated with resistance to docetaxel of breast cancer cells [41], but in Ewing’s sarcoma, miR-34a has been found to increase the sensitivity of cancer cells to doxorubicin and vincristine [42].

MiRNAs can regulate the disease progression through targeting mRNAs. miR-193a-5p can induce bladder cancer resistance to cisplatin by targeting AP-2α [43]; targeting of MiR-487a via BCRP/ABCG2 enhances the sensitivity of breast cancer cells (MCF7/MX) to mitoxantrone [44]; Studies have shown that MTDH can target and bind miR-26a-5p to play an important role in breast cancer and esophageal squamous cell carcinoma [45, 46]. However, this study showed that GEM had no significant effect on the expression of miR-26a-5p. miR-9-3p, which is closely related to the development of various tumors, plays different regulatory roles in different tumors through regulating different downstream target genes. Li et al found that miR-9-3p expression is up-regulated in non-small cell lung cancer cells, and this promotes proliferation and invasion of the cancer cells through activating AMP-related protein kinase pathways [36]; miR-9 targets MDK, and regulation of PDK/AKT pathway inhibits angiogenesis of nasopharyngeal carcinoma [47]; also miR-9-3p could inhibit the EMT of nasopharyngeal carcinoma cells through targeting FN1, ITGB1 and ITGAV [48]. To investigate whether miR-9-3p regulated the sensitivity of breast cancer cells to Gem through targeting MTDH, we examined the effects of low-expressed or overexpressed miR-9-3p and MTDH on migration, invasion, and EMT-related proteins of breast cancer cells. The data revealed that overexpression of miR-9-3p inhibited cell invasion and significantly promoted the inhibition effect of Gem on cell invasion, migration, and EMT, while overexpression of MTDH partially reversed such effects. Moreover, cell experiments with low-expressed miR-9-3p and silent MTDH also confirmed the results.

In conclusion, MTDH regulated the biological functions and drug resistance of Gem-treated breast cancer cells and affected tumor growth through targeting miR-9-3p.

## Supplementary information

Figure S1

Figure S2

Figure S3

Figure S4

Figure S5

Figure S6

Figure S7
