# Supplementary figures and images for: MiR-9-3p regulates the biological functions and drug resistance of gemcitabine-treated breast cancer cells and affects tumor growth through targeting MTDH
Source: Cell Death Dis. 2021 Sep 22;12(10):861. doi: 10.1038/s41419-021-04145-1 (PMC8458456; doi:10.1038/s41419-021-04145-1)

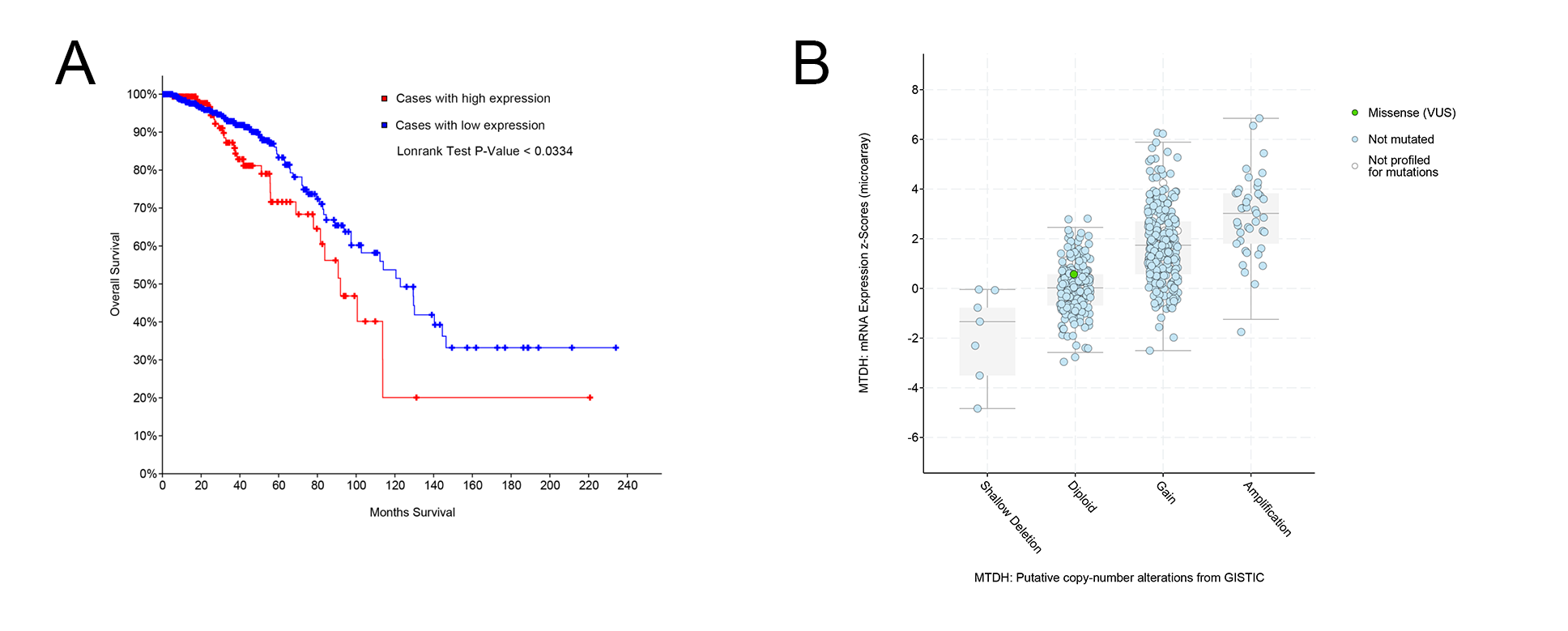

Supplement: Supplementary file 1 — Figure S1 [file 41419_2021_4145_MOESM1_ESM.tif]

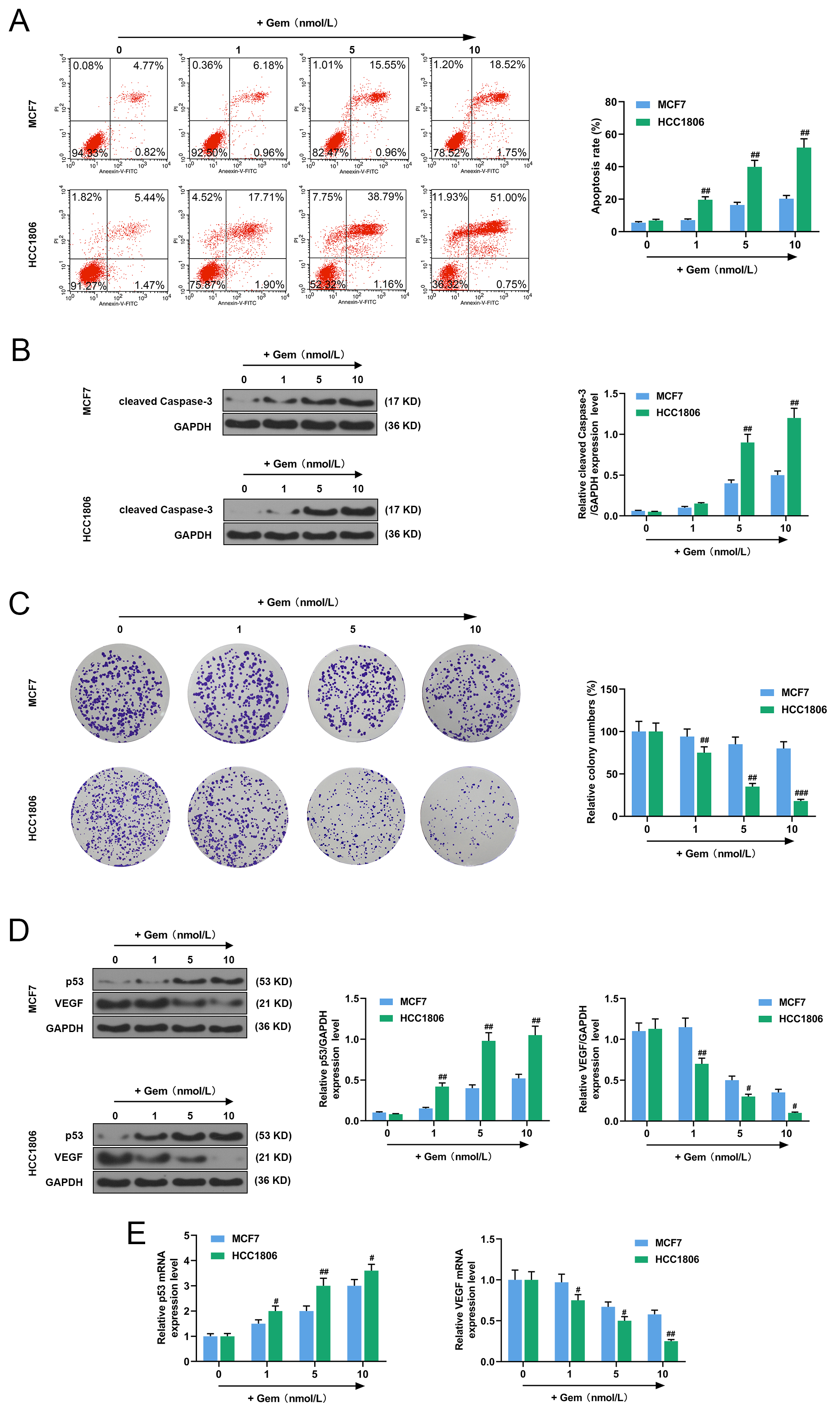

Supplement: Supplementary file 2 — Figure S2 [file 41419_2021_4145_MOESM2_ESM.tif]

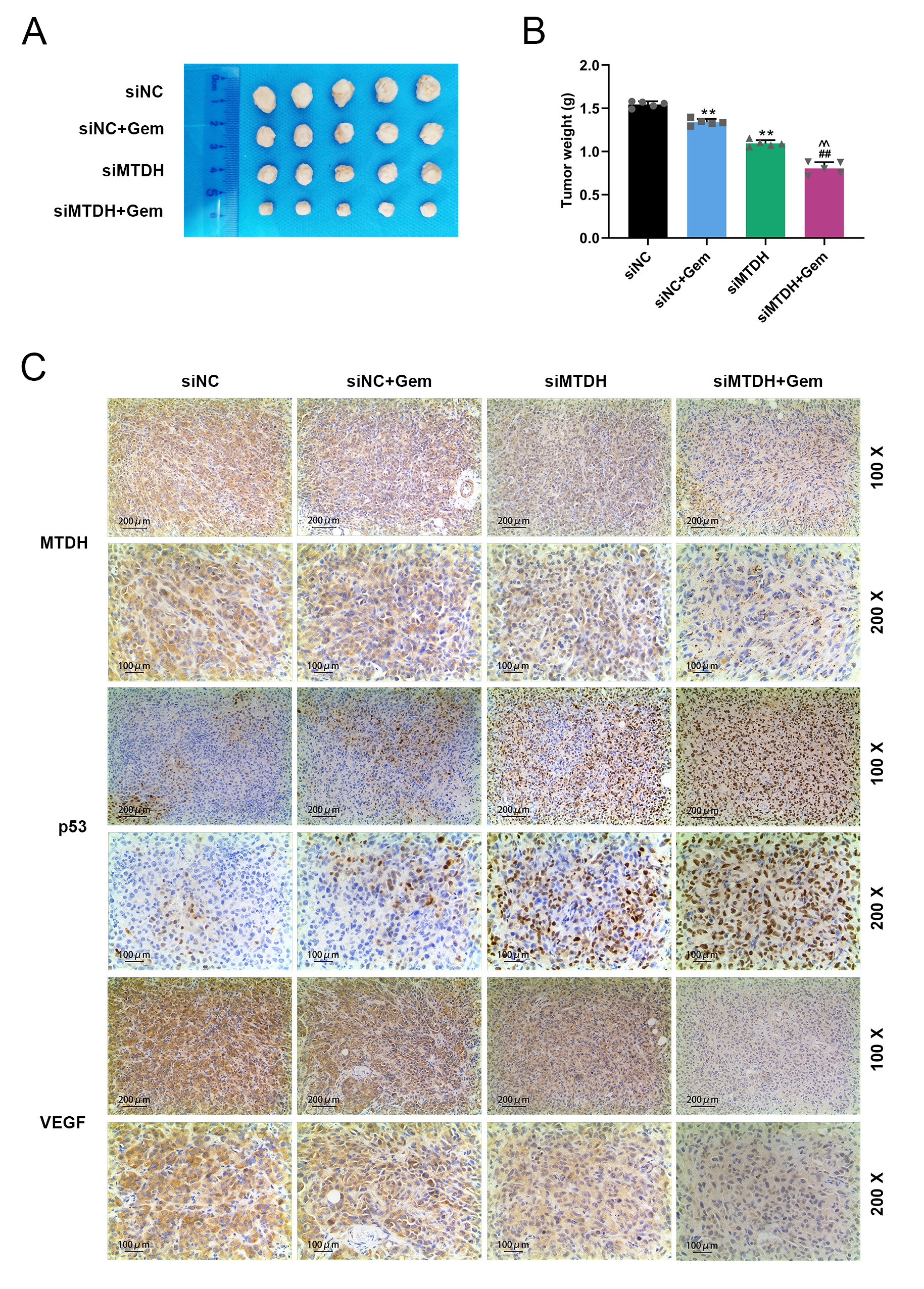

Supplement: Supplementary file 3 — Figure S3 [file 41419_2021_4145_MOESM3_ESM.tif]

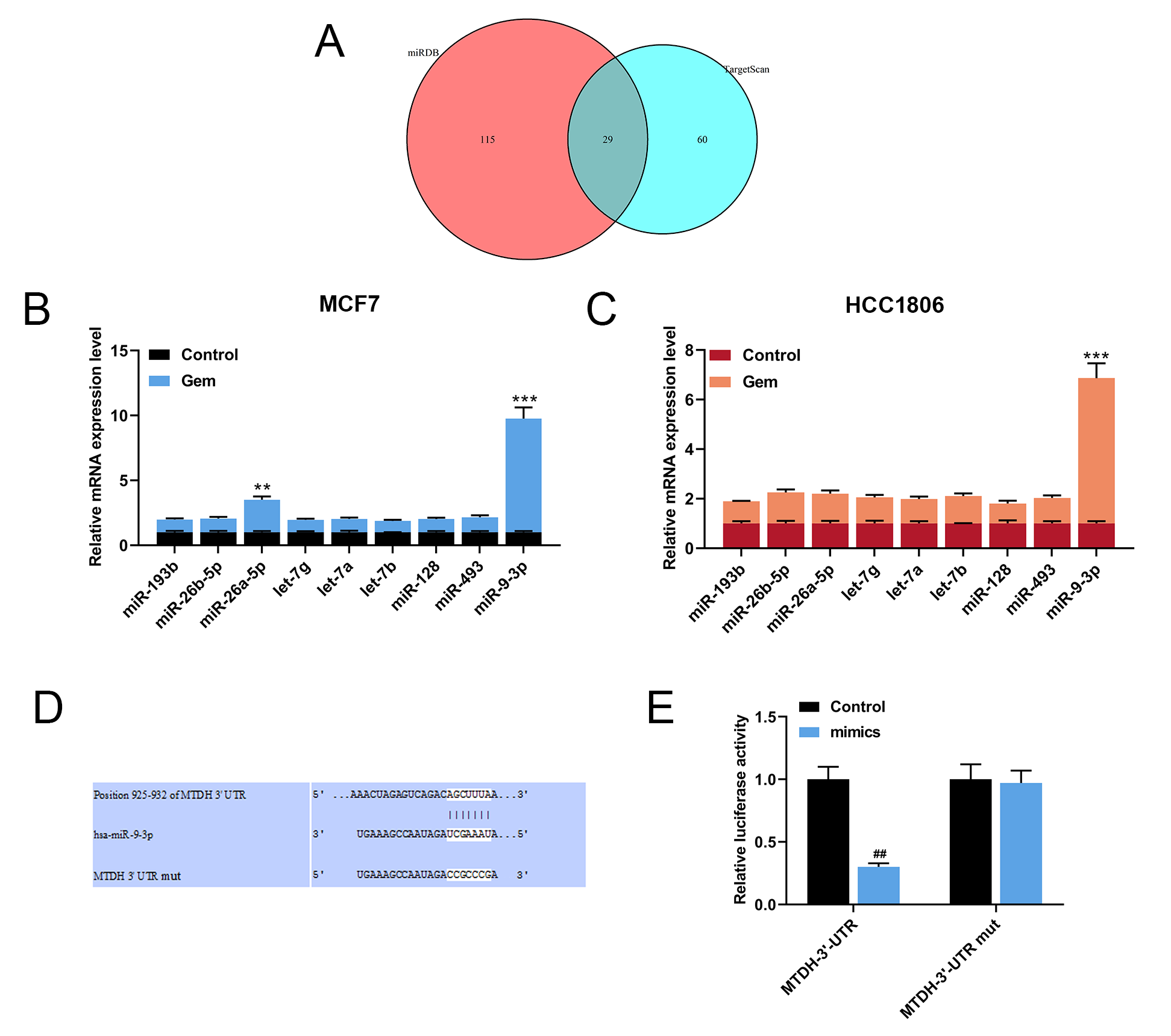

Supplement: Supplementary file 4 — Figure S4 [file 41419_2021_4145_MOESM4_ESM.tif]

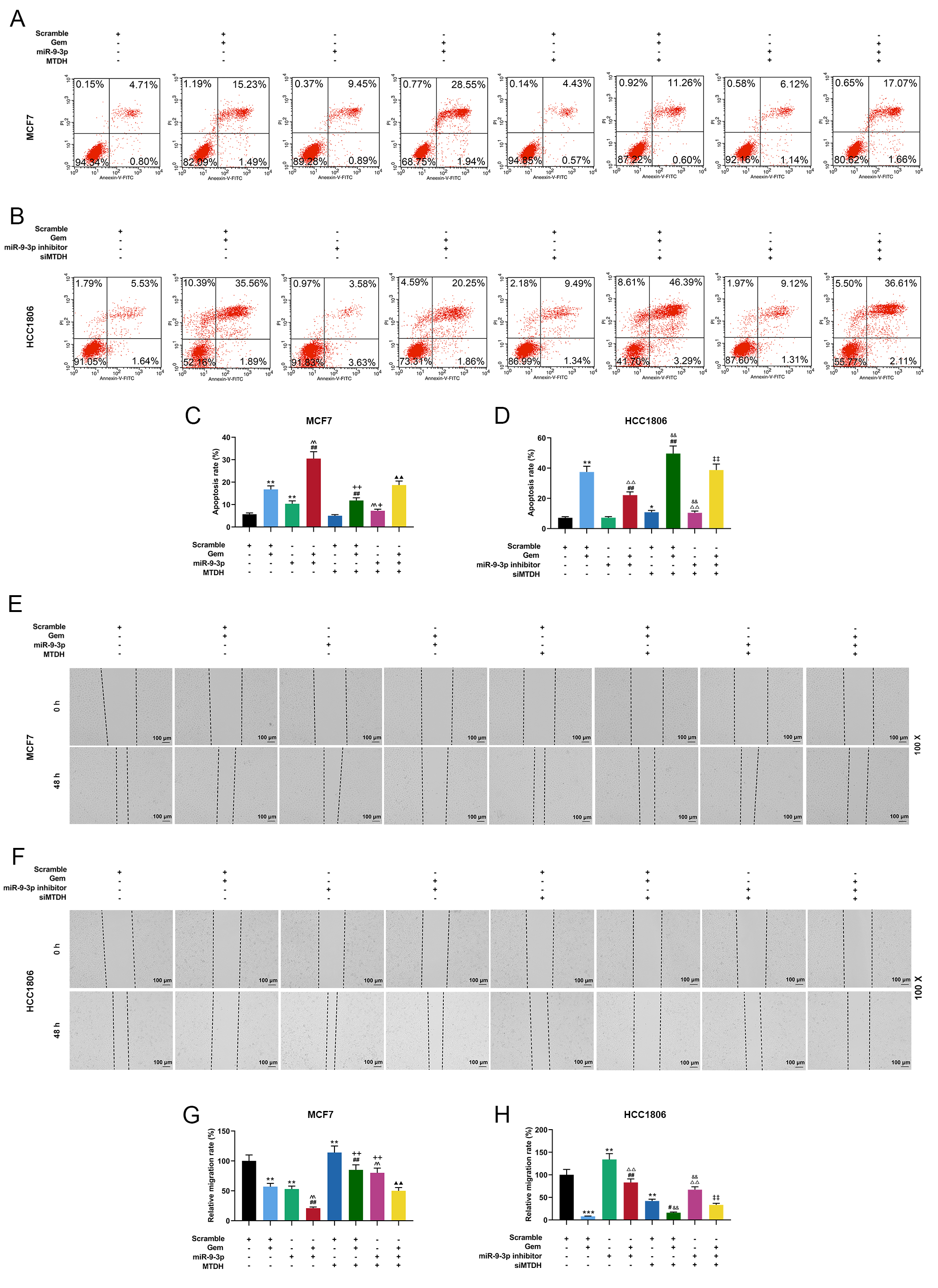

Supplement: Supplementary file 5 — Figure S5 [file 41419_2021_4145_MOESM5_ESM.tif]
