# Supplementary material for: MiR-9-3p regulates the biological functions and drug resistance of gemcitabine-treated breast cancer cells and affects tumor growth through targeting MTDH
Source: Cell Death Dis. 2021 Sep 22;12(10):861. doi: 10.1038/s41419-021-04145-1 (PMC8458456; doi:10.1038/s41419-021-04145-1)
Supplement: Supplementary file 6 — Figure S6 [file 41419_2021_4145_MOESM6_ESM.docx]

Table 1 Primers used in real-time PCR analysis

| Gene | Primer sequence | Species |
| --- | --- | --- |
| MTDH | Forward: 5'- AAATGGGCGGACTGTTGAAGT ‑3'  reverse: 5'- CTGTTTTGCACTGCTTTAGCAT ‑3', | human |
| p53 | Forward:5'- CAGCACATGACGGAGGTTGT-3'  reverse: 5'- TCATCCAAATACTCCACACGC-3' | human |
| VEGF | Forward:5'-GAACTGGGGGTACTGGGG-3'  reverse: 5'-TTCCCTAAGTGCTCCCAAA-3' | human |
| miR-193b | Forward: 5'- TGAGGGCGAGATGAGTCGTA ‑3'  reverse: 5'- TATCCAGTGCGTGCGTGTC ‑3' | human |
| miR-26b-5p | Forward: 5'- CGTATCCAGTGCAATTGCCG ‑3'  reverse: 5'- GTCGTATCCAGTGCGTGCG ‑3' | human |
| miR-26a-5p | Forward: 5'- GTAATCCAGGATAGGCTGTCGT ‑3'  reverse: 5'- TGTCGTGGAGTCGGCAATTG ‑3' | human |
| let-7g | Forward: 5'- ACAGTTGTCGTATCCAGTGCAA ‑3'  reverse: 5'- TATCCAGTGCGTGCGTGTC ‑3' | human |
| let-7a | Forward: 5'- AGTTGTCGTATCCAGTGCAA ‑3'  reverse: 5'- TATCCAGTGCGTGCGTGTC ‑3' | human |
| let-7b | Forward: 5'- TGGTTGTCGTATCCAGTGCAA ‑3'  reverse: 5'- TATCCAGTGCGTGCGTGTC ‑3' | human |
| miR-128 | Forward: 5'- CGTATCCAGTGCAATTGCCG‑3'  reverse: 5'- GTCGTATCCAGTGCGTGCG‑3' | human |
| miR-493 | Forward: 5'- CATGGTAGGCTTTCATTGTCGT‑3'  reverse: 5'- TGTCGTGGAGTCGGCAATTG‑3' | human |
| miR-9-3p | Forward: 5'- AAGTGTCGTATCCAGTGCAA ‑3'  reverse: 5'- TATCCAGTGCGTGCGTGTC ‑3' | human |
| U6 | Forward: 5'-TGACTTCCAAGTACCATCGCCA-3'  reverse: 5'-TTGTAGAGGTAGGTGTGCAGCAT-3' | human |
| GAPDH | Forward: 5'-GGTGAAGGTCGGAGTCAACG-3'  reverse: 5'-CAAAGTTGTCATGGATGTACC-3' | human |

Abbreviations: miR, miRNA; GAPDH: glyceraldehyde-3-phosphate dehydrogenase;
