# Supplementary material for: MiR-9-3p regulates the biological functions and drug resistance of gemcitabine-treated breast cancer cells and affects tumor growth through targeting MTDH
Source: Cell Death Dis. 2021 Sep 22;12(10):861. doi: 10.1038/s41419-021-04145-1 (PMC8458456; doi:10.1038/s41419-021-04145-1)
Supplement: Supplementary file 7 — Figure S7 [file 41419_2021_4145_MOESM7_ESM.docx]

vpnTable 2 Primary antibodies information of Western blotting

| Primary antibody | information |
| --- | --- |
| N-cadherin | rabbit, 1:1000, ab18203, Abcam, USA |
| cleaved-cysteinyl aspartate specific proteinase-3 (cleaved-caspase-3) | rabbit, 1:1000, ab2302, Abcam, USA |
| Caspase-3 | rabbit, 1:1000, ab32351, Abcam, USA |
| E-cadherin | rabbit, 1:1000, ab40772, Abcam, USA |
| Vimentin | rabbit, 1:1000, ab92547, Abcam, USA |
| p53 | mouse, 1:1000, ab26, Abcam, USA |
| MTDH | rabbit, 1:1000, ab45338, Abcam, USA |
| VEGF | mouse, 1:1000, ab1316, Abcam, USA |
| MMP-2 | rabbit, 1:1000, ab97779, Abcam, USA |
| MMP-9 | rabbit, 1:1000, ab73734, Abcam, USA |
| glyceraldehyde-3-phosphate dehydrogenase (anti-GAPDH) | mouse, ab8245, Abcam, USA |
